# Quantitative assessment of the repeatability of PermaGel as a ballistic tissue simulant

**DOI:** 10.1007/s00414-026-03821-1

**Published:** 2026-05-01

**Authors:** James Read, Samuel Yates, Rupert Williams, Rachael Hazael, Richard Critchley

**Affiliations:** Department of Engineering and Physical Sciences, Cranfield University, Defence Academy of the United Kingdom, Shrivenham, SN6 8LA UK

**Keywords:** Soft tissue analogue, Ballistic testing, Wound ballistics, Penetration, Repeatability

## Abstract

The awareness of economic, environmental and ethical considerations centred on replication of human tissue have increased in recent years. This has driven the need to find repeatable products which can be re-used without detriment to datasets whilst providing economic benefit to research programmes and reducing the quantity of material disposed of at the end of the trials phase. This study is focussed on the ability of PermaGel to provide a repeatable medium for both survivability and lethality applications when re-used continuously. A baseline series of tests were undertaken using a single stage gas gun, with the material samples then undergoing a series of melt/recast cycles before being tested again to examine any differences in performance. The results show that a 30.40% difference in depth of penetration was observed post thermal cycling of the material, whilst a 5.36% increase in the maximum diameter of the temporary cavity was also observed. These results have raised concerns on PermaGel’s ability to be re-used accurately and recommendations on its use provided to ensure data inaccuracy risk is minimised. The findings herein have confirmed the concerns raised within the literature, provided the research community with a single point of source data, and recommended areas for future work to ensure continued characterisation is undertaken.

## Introduction

Traditionally human cadavers and mammalian tissue have been used as a suitable medium to provide insight into ammunition performance, projectile lethality assessment, and wound ballistics studies [1–6]. However ethical concerns have been raised with the continued use of these materials, and alternative soft tissue analogues have been used in more recent times to ensure research programmes maintain an ethical focus throughout their duration [7–10]. To ensure compliance with ethical requirements, researchers have sought opportunities to utilise different materials that help to reduce their ethical impact without compromising datasets.

Ballistic gelatine has been widely adopted as both the industry and academic standard which has been reported to provide the closest replication of the human torso [5, 11–17]. Its ability to provide a transparent medium in which temporary and permanent cavitation can be analysed via high-speed video which is critical to ensuring improvements in survivability technology and ammunition development. Additionally, the importance of calibration has also been considered with the Fackler method being the industry approved method of ensuring a consistent medium is present within research studies when using 10% gelatine (water/weight (w/w)) [12, 14, 18–20]. The other most used ballistic gelatine variant 20% (w/w) is used within European and NATO countries as an increased density is required to test Full Metal Jacket (FMJ) rounds [11, 12, 19, 21, 22]. However, 20% gelatine is currently lacking a community approved methodology for calibration [11–13, 19, 23].

Whilst the positives of ballistic gelatine are clear, concerns within the academic literature exist, and are most notably raised in relation to temperature dependence, single shot use and manufacturing variability [11, 12, 19, 24–26]. Alternatives are therefore being sought to provide greater economic value to single shot surrogates, simulants that can be used within a broader range of climatic conditions and in more recent times, reducing the amount of material being disposed of enabling research programmes to move toward a more sustainable research practise utilising frameworks such as the 3R principle [12, 27–31].

Perma-Gel has been identified as a suitable alternative that could provide the research community with a viable solution.

Perma-Gel is a synthetic ballistic gelatine substitute mainly used within forensic science, ballistics testing, and medical research [11, 12, 32–34]. Perma-Gel aims to simulate human soft tissue and enable researchers, medical professionals and wider fields to investigate penetration depth, observe temporary and permanent wound cavities, evaluate projectile or fragmentation performance, and to directly compare performance of different bullets or calibres [11, 12, 24, 32, 34, 35]. Manufactured using gellants and mineral oil, this material has been reported to serve the same purpose as traditional 10% ballistic gelatine when evaluating terminal ballistics (i.e. what happens when the bullet or projectile hits a target) but removes the reported limitations [12, 24, 36].

Perma-Gel has been reported to provide additional benefits to traditional Ballistic Gelatine, primarily its ability to be re-used multiple times, removing the need to purchase high volumes of material thereby reducing economic and supply chain burden during testing [37, 38]. This is achieved by melting the material and recasting into the required sample profile. Care and consideration should be given during this process to ensure the material does not degrade - a phenomenon for which the academic literature has reported and has been found to contain numerous conflicting views and findings specifically focussed on the materials ability to continue performing advantageously after repeated melt cycles [12, 24, 33, 39]. Additionally, benefits such as consistency and reproducibility in simulating soft tissue properties are also noted alongside enhanced transparency allowing for visualisation of projectile trajectory inside the media, penetration depth assessment and witnessing cavitation dynamics are also noted [11, 24, 32, 35]. Stability over time compared to ballistic gelatine is also key with no requirement to keep the material refrigerated before testing and greater ability to utilise the material in a wider range of environmental conditions. This attribute is crucial in ensuring a longer lifespan of the material which minimises the probability of suffering from the mould growth and malodorous smell emitted from traditional ballistic gelatine in uncontrolled environments [40–42]. Perma-Gel is delivered in a manufactured state and is ‘ready to use’ having been calibrated to the FBI standard for 10% Ballistic Gelatine at 4 °C, enabling users to link directly to a referenceable source which is both well-known and well understood [43, 44].

Several limitations and critiques of Perma-Gel have been identified and are important to highlight, namely the degradation of mechanical properties caused by thermal cycling (reporting to be a benefit of Perma-Gel) [11, 12, 15, 24, 32]. Repeated thermal cycling of polymeric materials can induce chemical or physical degradation, potentially altering key properties such as elasticity or density (thereby impacting a full assessment of penetration depth and wound profile) [45–47]. For materials like perma-gel, such changes may have important implications for its ability to reliably simulate tissue response in ballistic events, and therefore its applicability in specialisms such as wound ballistics, survivability assessment, lethality and ammunition design, and forensic reconstruction. Additionally, problems occurring during thermal cycling such as air bubble formation (leading to inconsistencies in density and structural integrity), uneven heating (localised degradation or incomplete melting resulting in non-uniformity in material samples), contamination (dust, debris or residue from previous uses) and the potential loss in material (from evaporation, spillage, or residue left within moulds resulting in differences in final sample volume and consistency) can impact the accuracy of test results.

Differences in mechanical properties compared to human tissue (i.e. elasticity, density, or failure modes) are also of concern. Strain rate and mechanical equivalence of Perma-Gel are reported to behave between 10% and 20% Ballistic Gelatine variants, with potential differences in retarding force/exit velocities than human tissue [11, 32, 34]. The literature has reported higher exit velocities in Perma-Gel when compared to other soft tissue surrogate alternatives, which may suggest less retarding force than standard gelatine under specific conditions [15]. This is important to understand to ensure penetration of projectiles are full understood and not overestimated. These differences in mechanical behaviour are important to note as without sufficient control, mapping to tissue behaviour directly may not be exact.

Differences in material properties are often controlled via use of Calibration. Calibration to the FBI standard at 4 °C has previously been reported as being of benefit to Perma-Gel, however some concern exists regarding is appropriateness given the differences in material density. The literature is widely populated when using this procedure for traditional ballistic gelatines, yet paucity of literature exists on this procedure’s applicability to Perma-Gel and its findings on how well this material remains calibrated throughout its life when considering thermal cycling and prolonged exposure to environmental conditions without sufficient refrigeration.

Economic and sustainability should also be considered. Perma-Gel has been found to have a greater upfront economic impact to a research programme, however by re-melting and recasting the material can provide savings throughout the research programmes duration [15]. The authors have also noted potential concern regarding the disposal of the material. Unlike traditional ballistic gelatine, Perma-Gel contains mineral oil, which is reported to not be bio-degradable [48, 49]. Should incorrect post processing and disposal of the material take place, this could have a negative impact on the environment and each research establishments sustainable goals [12, 15, 50–52].

Noting the above limitations, it would suggest that Perma-Gel is best suited to being used comparatively rather than assuming absolute fidelity to human tissue and must include calibration or validation steps if possible. Researchers should track and limit re-use of blocks, documenting any changes in appearance (colour, clarity, bubbles, voids) and mechanical responses where possible to ensure any differences do not negatively impact the output data. Additionally, consideration should be given to maintaining consistent environmental conditions such as temperature, block size, orientation, and backing support to minimise variability and acknowledge that the results (temporary cavity size, material displacement, and fragmentation behaviour) may not directly translate into human wound outcomes for which appropriate caveats should be included in the research discussion.

Consideration of the materials limitations is important, but so is the comparability with existing Soft Tissue Surrogates. PermaGel has most commonly been used within the wider literature to replicate the aims of Ballistic Gelatine [11, 12, 15, 21, 24, 32, 33, 53, 54]. To articulate the benefits of PermaGel compared to the industry standard, Table 1 highlights they key features deemed important within wound ballistics and testing and the differences between both materials.

**Table 1:** PermaGel and Ballistic gelatine comparison

Feature/Attribute	Ballistic Gelatine	Perma-Gel
Material	- Gelatine made from animal collagen diluted to 10% or 20% by mass [11, 12]	- Polymer/synthetic gel formulated to mimic soft tissue [34, 36]
Primary Advantage	- Industry standard (historic use) [12, 43] - Well understood baseline	- Reusable, [24, 39] - Shelf-stable, [24, 39] - Consistent across batches. [24, 39]
Clarity/Visibility	- Clear-ish but can be cloudy [12, 15] - Visibility decreases with re-use (hence single shot preference)	- Very Clear – excellent for observing wound tracks and formation [12, 24, 39].
Temperature/Storage	- Must be refrigerated and used fresh [12, 18, 19].	- Stable at room temperature - Long Shelf Life [24, 39].
Reusability	- Traditionally used a single shot use material due to issue regarding clarity.	- Designed to melted and reused many times [24, 39].
Batch Consistency	- Medium – Standardised procedures are available, however risk of incremental differences during manufacture [19].	- High (factory formulated for uniformity)
Calibration	- Calibration procedures available for 10% variant (Fackler) [12, 43]. - No industry accepted calibration procedure for 20% variant [12]	- Manufacturer supplies procedure to match 10% gelatine reference [44].
Behavioural Differences	- Good replication of average soft-tissue response (Industry Standard) [11, 12]	- Good replication of average soft-tissue response [24, 34, 39] - Projectiles may behave differently vs. organic gelatine [34]
Preparation	- Mix and cool to set [14, 15, 18, 19, 55] - Susceptible to spoilage and mould if stored incorrectly [18, 19]	- Melt/recast and pour as directed [24] - Minimal spoilage
Cost	- Lower per block cost but reoccurring expense and logistics [15].	- Higher initial cost, but lower long term due to reuse [12, 15]
Practical Lab Needs	- Refrigeration/freezer and more lab preparation and disposal required [12, 15, 19]	- Oven or heat source to melt/recast [24] - Less cold storage
Health/Sanitation	- Biological material requires hygienic handling and disposal	- Low bio-hazard risk - No rotting/smell
Limitations	- Homogenous substitute – no skin, bone, layered tissue or vascular perfusion^1^ - Variability from organic material.	- Homogenous substitute – no skin, bone, layered tissue or vascular perfusion

^1^& 2 Layering of simulants to provide a representative human model has been reported here as a point of interest within the research community, however further discussion has been negated from this work due to limitations in scope

Although Table 1 has highlighted that PermaGel is akin to Ballistic Gelatine, differences do exist and therefore use of this material should be undertaken with caution. Perma-Gel is marketed as being equivalent to 10% gelatine, but the literature indicates that its mechanical response may differ in high strain rate environments [21, 34, 54]. Additionally, previous studies have conflicting views on how many cycles of melting and re-casting are available to the user before material degradation begins [12, 24, 33]. Boackle et al. have reported a that Perma-Gel can be reused up to 12 times, however this is contradicted by discussions between Tischler and Mabbott who have cited 10–15 times [24, 33]. Such inconsistencies within open literature make the decision on reusability challenging and pose additional risk of inaccuracies within datasets.

This study aims to provide the academic literature with a single point of source data that provides both academia and industry with a clear understanding of the number of melting cycles required before degradation becomes an issue, and an assessment of the degradation severity within a ballistics setting. By assessing changes in penetration depth material consistency, and elasticity across multiple re use cycles, the authors aim to determine the practical limitations of Perma-gel as a reusable wound ballistics simulant.

## Materials and methods

### Materials

Moulds of dimension 9” x 4” x 4” (228.6 mm x 101.6 mm x 101.6 mm) (+/- 0.25 mm) were manufactured by the Cranfield University workshops using Aluminium. Aluminium was selected as the material of choice primarily due to is proven ability to act successfully as a mould for this type of application [56, 57], its workability, and ability to provide an economical solution to this studies requirement. To ensure transparency of the material was maximise, consideration of surface sidewall damage during decanting was given, and minimised by ensuing as much of the mould was manufactured from a single sheet of Aluminium before being welded to create a watertight seal. Additionally, the application of Liquid Wax release agent was utilised to reduce the potential of adhesion and therefore damage during decanting.

**Fig. 1:**
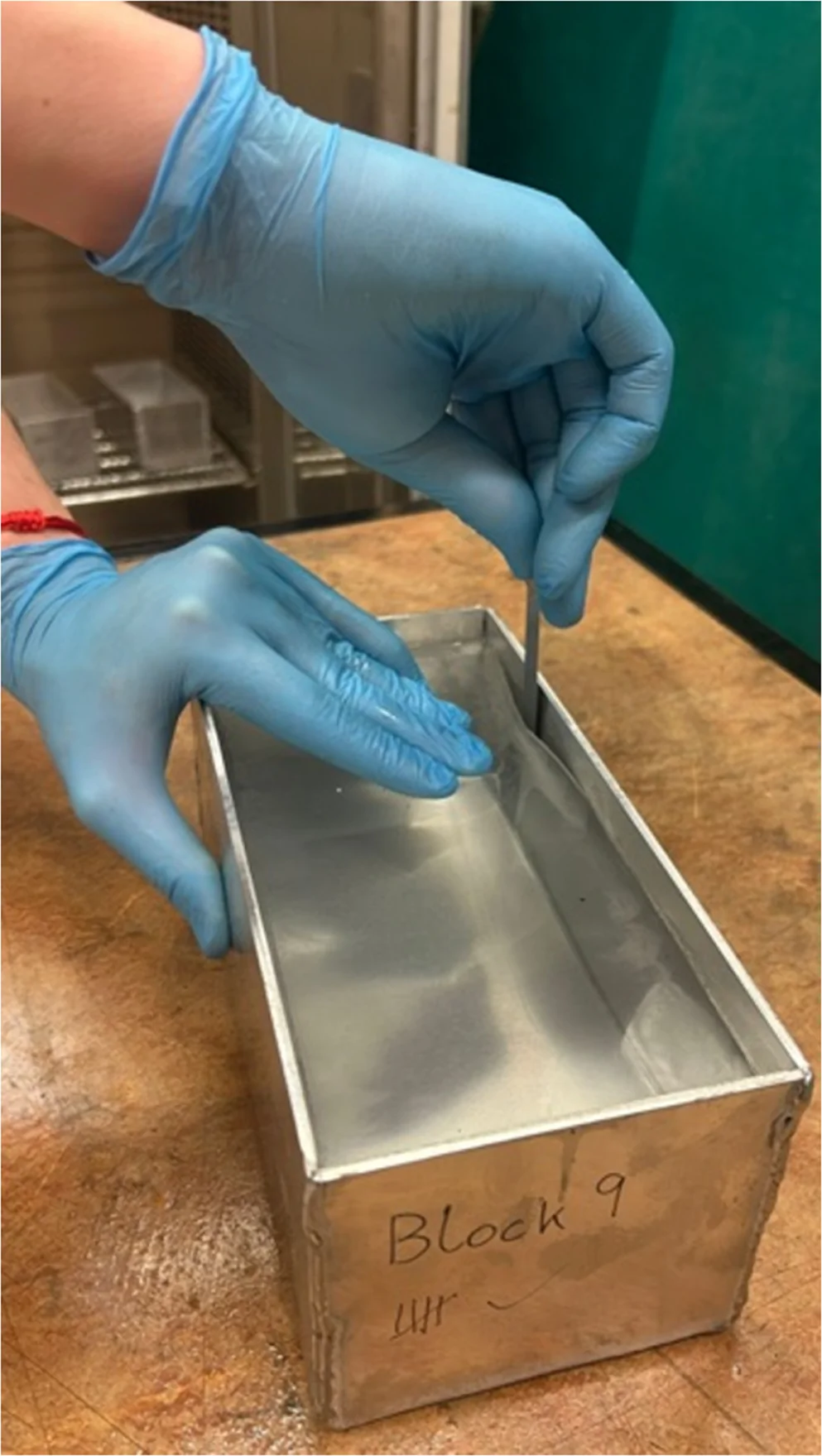
Perma-Gel material extraction post cure

The Perma-Gel material was supplied whole and stored before use, before being shot once per block, before being exposed to its melting/re-casting cycle. Post melt, all samples within this study were individually poured into the moulds before being left to solidify at room temperature (18 °C) to set. Once each mould contained a solidified material sample that had undergone its designated melt and re-cast cycle, a surgical scalpel was used to ensure any cohesion between the material and mould walls were reduced before being carefully extracted (Fig. 1).

### Perma-Gel

10 Blocks of Perma-Gel were purchased from Ballistic Dummy Lab [44] at a cost of $69.99 per block^2^.

**Fig. 2:**
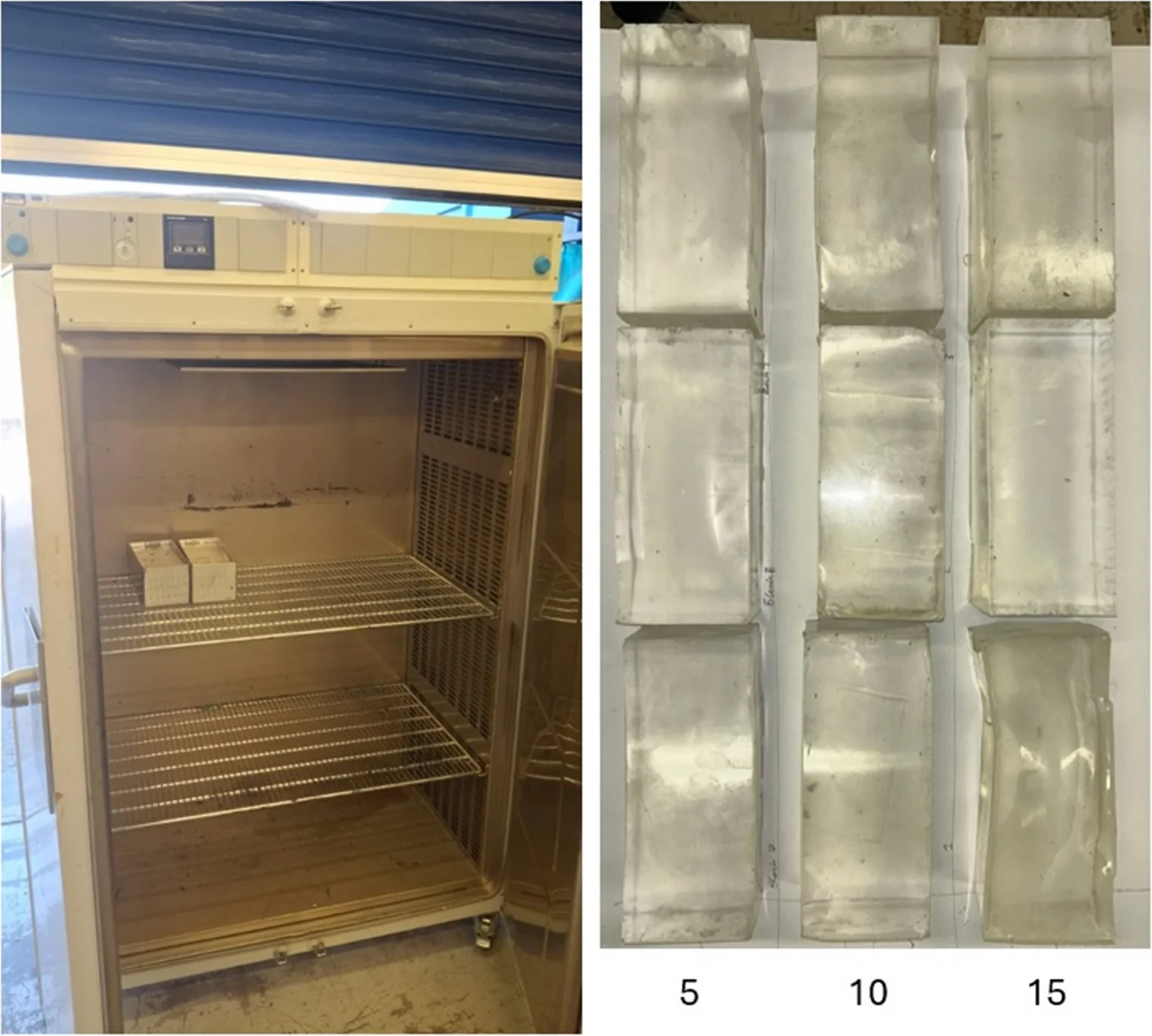
Left: Heraeus oven. Right: Material condition post artificial ageing arranged by number of melt cycles (Yellowing shown bottom right sample)

As this study is primarily focussed on the mechanical performance of Perma-Gel when subjected to thermal cycling, a Heraeus D-6450 Hanau Oven (Fig. 2) was pre-heated to 117 °C which was ascertained via a calibration test prior to this study and consistent with other studies and technical notes associated with the same material [24, 37]. A series of procedures were followed to ensure the material was melted under controlled conditions, before being allowed to cure at a consistent temperature. Table 2 articulates the conditions and observations.

**Table 2:** Perma-Gel manufacturing conditions and observations

Number of Samples	Oven Temperature (°C)	Cure Temperature (°C)	Melt/Recast Cycles	Observations
3	117 for 3 h	18 for 5 h	5	N/A
3	117 for 3 h	18 for 5 h	10	N/A
3	117 for 3 h	18 for 5 h	15	Yellowing present on 1 x material sample after thermal cycling.

### Dynamic testing projectile

AISI 420 grade stainless steel ball bearings of 4.5 mm diameter were integrated to a pre-stressed plastic sabots to ensure no energy would be lost during firing from air passing around the projectile in the gun barrel [14, 15]. The sabots had been pre-stressed to ensure projectile separation prior to material impact and minimise any risk of impeding the visual inspection of the projectile entry site. Each ball bearing, with a mass of 0.4 g, underwent a visual inspection before being inserted into the gas gun breech.

## Set up and method

### Dynamic testing

The ELVIS Gan Gun located at Cranfield University Shrivenham campus was used to conduct the experiments for this study. Full details of this gas gun and associated equipment used for this set up can be found within previous works by the same authors [14, 15].

The ball bearing was integrated to a plastic sabot, before being placed within the gun breach and closed. The High-Speed Video (HSV) camera and internal high-powered light were turned on prior to being gas gun being pressured to 12 bar of air (+/- 1 bar). Two light gates were used to accurately record the arrival time of the projectile and proven to deliver the projectile to the target at 235 ms^− 1^ (+/- 12 ms^− 1^). This velocity was chosen to align to previous Perma-Gel testing undertaken by A.Mabbott within the same research centre, whilst also considering the limitations on the Gas Gun [24]. Once pressurised, the gas gun was fired safely from a remote location. It should be noted that during the second stage of firings, the air used to run the Gas Gun was unavailable and therefore 8 Bar of Helium was used to achieve the same velocities.

Post firing, each sample was removed from the target chamber before being visually inspected for entry witness marks and permanent cavitation with observations noted. Where observations had been noted, these were subsequently validated using the HSV footage. Measurements of depth of penetration were undertaken using a steel rule which were later confirmed using the HSV footage to ensure no projectile withdrawal had taken place during temporary cavity collapse (by the rapid evacuation of air during elastic recovery), thereby falsifying the dataset. Based on repeat measurements by different operators, the uncertainty in the penetration measurement for this study is on the order of 0.5 mm.

The HSV camera used within this study was noted as a Phantom V12-12 with the settings provided in Table 3. This camera was used alongside the associated PCC software (version 3.11.11.806) to analyse projectile/material interaction including the measurement of projectile travel, wound cavity diameter, formation and collapse, shock wave transmittance, and any other observations of interest. To ensure this was done accurately, the camera was calibrated and values being noted (Table 3) to ensure appropriate scale setting was entered during post processing of the images and video. Additional post processing of the raw dataset outside of the PCC software was conducted using Microsoft Excel.

**Table 3:** Phantom High-speed camera V12-12 settings

Phantom V12-12
Resolution = 1024 × 352
Frames Per Second = 33,000 fps
Exposure = 5.00µs
Calibration = 0.368 mm/pixel (Serial 1) Calibration = 0.363 mm/pixel (Serial 2)

Two serials were undertaken to generate data for this manuscript. Serial one was primarily focussed on creating a baseline understanding of the materials performance by investigating the projectile depth of penetration using the material ‘as delivered’, whilst the second serial focussed on assessment of the changes to depth of penetration caused by varying melt and re-cast cycles.

## Results and Discussion

### Depth of penetration assessment

**Fig. 3:**
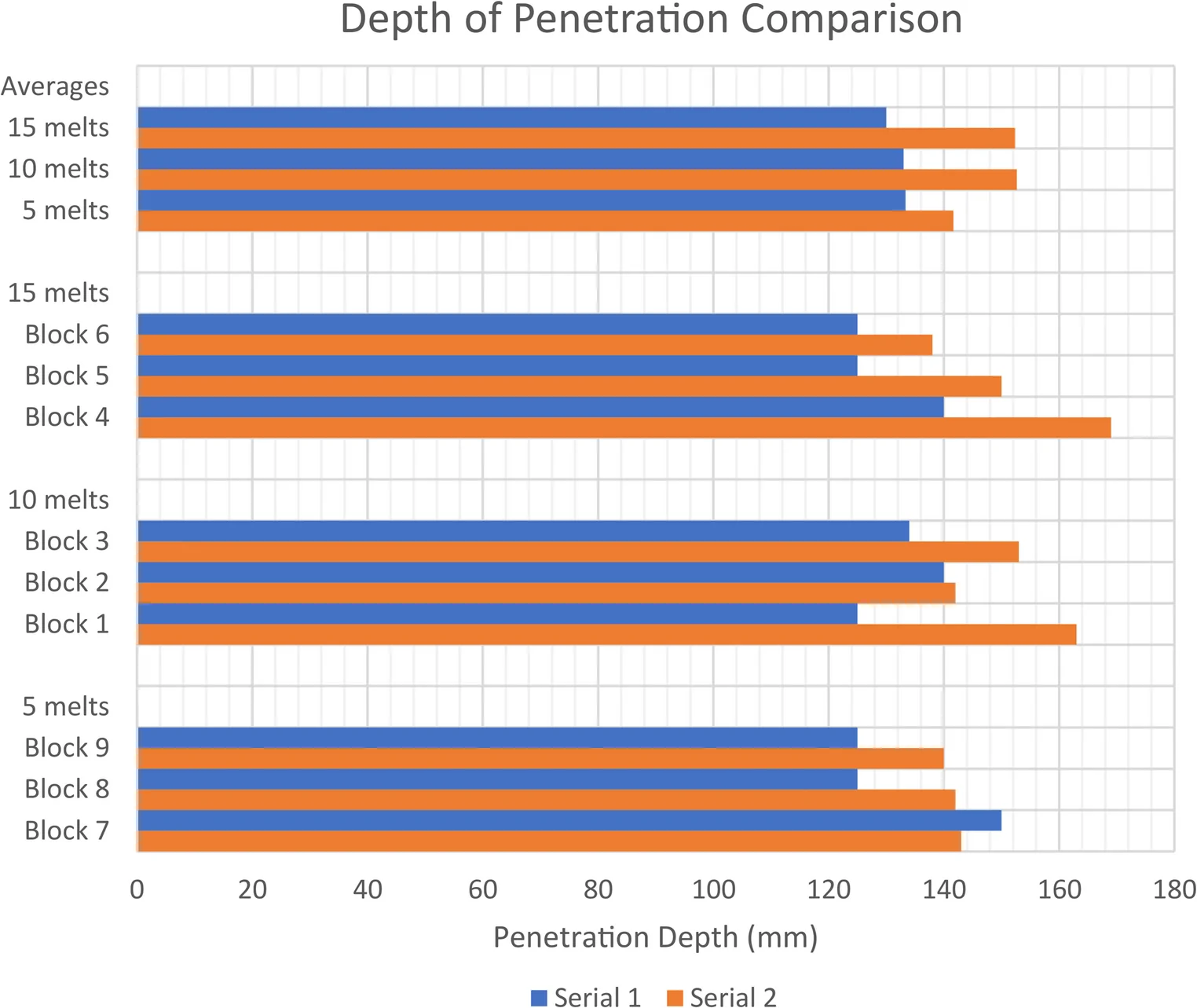
Perma-Gel Depth of penetration comparison between as-supplied material (Serial 1) and material which has undergone thermal cycling(Serial 2) in order of severity

Figure 3 shows the depth of penetration for the ‘as received’ material (serial one) and samples which has been subjected thermal cycling (serial 2). Across all samples used during serial one, the depth of penetration remains within a range of between 130 and 155 mm (17.54% difference). The velocity during baseline firings equated to an average of 239.1 ms^− 1^ with a variance of 8.88% between the lowest (226 ms^− 1^) and highest (247 ms^− 1^) velocities recorded. Comparison of the velocity and depth of penetration raw data shows non-linear trends (Annex A – Depth of Penetration and Velocity Raw Data), highlighting no correlation between speed and depth of penetration measurement, as evidenced by the lowest velocity recording (Block 1–132 mm) and the highest velocity recording (Block 6–133 mm). The raw data instead highlights an input velocity of 245 ms^− 1^ resulted in the highest depth of penetration measured at 155 mm, whereas the lowest depth of penetration (129 mm) was achieved at 241 ms^− 1^ providing numerical evidence of concerns regarding ‘as supplied’ material calibration and/or homogeneity.

On completion of the recorded baseline, an assessment of the change in depth of penetration measurement as a result of melt and re-cast was undertaken. Figure 3 shows that after 5 melts an average change of 9 mm (6.25%) was witnessed, whilst 10 and 15 melt cycles provided average results of 20 mm (14.79%) and 22 mm (17.18%) respectively when compared to the as supplied material depth of penetration values. These findings highlight that the material is no longer behaving like the original after being melted and as a result, may produce falsified data. The penetration depth of viscoelastic materials is traditionally governed by mechanical properties such as density, elasticity, viscosity, yield strength and resistance to deformation [2, 11, 14, 15, 23, 25, 58–60], a 12.74% increase suggests that the material has become less resistant (softer). It is also noted that a 12.74% increase in wound ballistics is beyond ‘typical’ experimental tolerances which are referenced within the widely accepted Fackler 10% gelatine calibration method which explicitly states +/- 0.5 cm to the 8.5 cm depth of penetration calibration value [14, 18, 19, 43]. Concerns are therefore raised with the materials ability to withstand higher volumes of melt cycles for repeatable experimentation.

### Cavitation depth and diameter differences

To further investigate the influence melting and recasting the samples had on the materials ability to provide the user with reliable source data, a comparison of pre and post melt cycle maximum temporary cavity diameter was conducted. This area of investigation was chosen primarily due to the temporary cavity’s importance in the assessment of energy transfer from projectile to tissue simulant and how that energy can damage structures beyond that of the immediate projectile pathway (permanent cavity) [61–69].

The temporary cavity has been proven to provide the most accurate real-world indicator of the amount and rate of energy that a projectile transfers into the material, as evidenced by high strain rate impacts resulting in greater energy transfer and therefore larger temporary cavities. This provides the user with an assessment on the potential for secondary indirect injuries through stretching or tearing of tissue commonly associated with organs, vessels and nerves [64–69]. Further, the temporary cavity assessment provides the user with increased data fidelity when compared to assessment of entry wounds alone. Assessment of entry wounds remains important, but different types or calibres of ammunition may result in very similar appearance on the sample surface, however the severity within the material is often influenced by velocity, projectile design (fragmenting, tumbling etc.), and stability during flight to, and travel within the sample [67, 70–72]. The outcomes of the ability to assess temporary cavity dynamics ultimately influences enhancements to projectile design, medical treatment and the ability to replicate forensic crime scenes where penetration or perforation of the human body as occurred [11, 64, 67–69, 73].

It is therefore vital to understand any potential impact on the ability for Perma-Gel to accurately provide users with an assessment on the temporary cavity which include not only material performance but also its ability to maintain transparency throughout the experimental procedure.

Table 4 highlights the values recorded during both depth of penetration and cavitation assessment. Assessment of the average cavity diameter data provides averages of 13.63 mm, 12.15 mm and 14.55 mm during baseline firings for the samples identified for 5, 10 and 15 melt cycles respectively. When compared to the post melt values of 13.42 mm, 14.62 mm and 14.50 mm this data provides evidence that an average increase of 5.36% in average cavity diameter after melting highlights a reduced reliability and repeatability of the material, this is an important finding when considering energy-transfer as small differences in material performance will result in later tests within the same series no longer reflecting earlier tests. This therefore results in reduced comparability between studies which may result in misinterpreted forensic interpretation, indirect comparisons between ammunition performance (where ammunition may appear to be more damaging), and ultimately mislead manufacturers to either falsely claim compliance with design requirements or focus on material enhancement in areas which are not required.

**Table 4:** Perma-Gel depth of penetration (DoP) measurements

Number of Melts	Sample No.	Serial 1 (mm)	Serial 2 (mm)	Difference between Serial 1 and Serial 2 DoP (%)
5				
	Block 7	150	143	−4.67
	Block 8	125	142	13.60
	Block 9	125	140	12.00
10				
	Block 1	125	163	30.40
	Block 2	140	142	1.43
	Block 3	134	153	14.18
15				
	Block 4	140	169	20.71
	Block 5	125	150	20.00
	Block 6	125	138	10.40

Despite the 5.36% increase being a small difference when reviewing the raw data, it does provide concern that the long-term use of Perma-Gel undergoing melting and re-casting within the conditions used in this study does result in deterioration of the mechanical properties. The data suggests that the material has become more elastic meaning that the material tends to overpredict the temporary cavity as the number of melt cycles increases. It is therefore recommended that during future use of this material this finding be factored into data error to mitigate against misleading findings being published within the wider academic literature. Additionally, the number of melt cycles is recorded to capture any degradation in performance.

To further investigate this finding, an assessment of the depth at which the temporary cavity maximum diameter occurred was undertaken to further contextualise its impact. In this assessment, pre-melt samples provided average depth of 61.12 mm, 60.26 mm and 63.46 mm (samples identified for 5, 10 and 15 melt cycles respectively) using factory supplied material versus the 67.91 mm, 53.44 mm, and 57.88 mm measurements for 5, 10 and 15 melt cycles respectively. These findings highlight a non-linear change relative to the number of heating cycles which is hypothesised to be due varying severities of molecular change during heating, but it is also noted that variance in input velocity may have influenced these findings. Additionally, this finding further emphasises that the materials mechanical performance has changed when compared to the original factory supplied blocks and provides further evidence that the melt-cycles have altered the polymer structure reducing in a material which is slightly softer (reduced yield strength) which also raises concerns around maintained use of a calibrated test medium during a prolonged test series.

When reviewing the findings surrounding maximum temporary cavity diameter changes post melt-cycle, it is recommended that a limit be placed on the number of times Perma-Gel can be melted and recast before disposal is required to ensure reliable and repeatable findings are produced and data inaccuracy within the open literature is minimised.

### Material use

This study has reported that the effects of melt/recasting cycles have a detrimental impact on Perma-Gels performance, however some additional concerns with the materials use have been noted. The literature does not currently provide detailed review of the materials constituent parts and their role in enabling the material to perform advantageously (exact polymer type, crosslink density, fillers and stabilisers etc.). This is important to understand as small changes in polymer mixes can dramatically change the materials viscoelastic properties (elasticity), yield stress and damping rate which all contribute to the measurement of both permanent and temporary cavity, and depth of penetration measurement [15, 74–79]. Additionally, in most cases, the material is used ‘as supplied’ and no verification of manufacturers claims appears to be undertaken prior to use in experimental campaigns [24, 34, 35, 39]. This introduces risk into test programmes by not establishing a baseline. Mitigation is provided in the form of a calibration certificate from the supplier stating that the batch of material supplied has been evidenced to conform to the FBI protocol, however from a researcher prospective assumptions must then be used including those concerned with material quality control, homogeneity, and polymer crosslinking consistency which can be summarised under ‘batch to batch variation’.

Inconsistencies in polymer crosslinking (main driver in controlling elasticity and energy dissipation) have been reported to result in variable stiffness and failure behaviour of other gelatines used within this field of study and should therefore be monitored throughout test to ensure molecular breakdown is not attributing to inaccuracies in data [80–83]. Additionally, lack of universally accepted calibration or lot-certification for synthetic gels may lead to material being received which is visually and identifiably identical but have differences in mechanical responses leading to inconsistencies within the test series and potentially across labs [12, 43, 53].

Concerns were also held during the process of melting and recasting of each sample which have been noted during this study, most notably thermal degradation of the polymer, incomplete homogenisation, microbubbles, material contamination and loss of clarity.

Investigation of the effects of thermal degradation of Perma-Gel was the primary aim for this study and has reported that under the test conditions used in this study, the data provided by this work confirms the hypothesis that increased numbers of melting cycles may detrimentally impact the performance of the material [12, 24, 33, 39]. The initial hypothesis centred on the logic that polymer chains are often susceptible to heat causing chain-scission), resulting in reduced elastic modulus and tensile strength [84–86]. Whilst this is an important consideration, thought must also be given to the degradation of plasticisers and stabilisers within the material which may have caused shifts in viscosity resulting in differences in mechanical performance, with the findings within this study pointing toward changes to viscosity [87–89]. This work, whilst providing an introductory understanding to the effects of thermal cycling on Perma-Gel is not without its limitations. The primary limitation of this study is that no physical testing occurred between heating cycles which may provide additional understanding on the degradation mechanisms and its severity. Additionally, it also remains unclear whether the physical work done during impact of a projectile impacts the rate of degradation when paired with thermal cycling.

Optical clarity was also monitored during the melt/recast cycles via visual inspection to ensure no micro-bubbles were being introduced to the material thereby being a detriment to the ability to examine high-speed video footage, but also introducing the potential for premature material failure due to localised weak spots [14]. During the manufacture of samples and pre/post-test visual inspection no micro-bubbles were found the be present. However, slight yellowing to one sample which had undergone 15 melt-recast cycles was apparent which is predicted to be as a result of oxidation and breakdown of stabilisers and plasticisers within the material resulting in discolouration (Fig. 2) [88, 90, 91]. This phenomenon was not apparent on the other two samples which underwent the same amount of cycling and were exposed to the same conditions, thereby confirming the concerns surrounding homogeneity of the material during the melt/recast process and the potential for mechanical drift . .

During the melt and recast process, it was observed that moderate quantities of fumes were emitted from the blocks, which did not reduce with the amount of cycles it underwent potentially linking the findings listed above to the marginal decreases in performance presented in Table 5. The fumes were cloudy in nature presenting an opaque visual reference and was acrid in smell, which required the oven to be relocated to an area with increased ventilation and additional personal protective equipment utilised to mitigate against inhalation. It is recommended that this observation be noted and considered during future works to ensure experimentation is conducted safely.

**Table 5:** Perma-Gel cavitation max. Diameter and Depth comparison Pre and Post melt cycle

Number of Melt Cycles (Block No.)	Average Input Velocity Pre Melt (m/s)	Pre-Melt Temp Cavity Depth (mm)	Pre-Melt Temp Cavity Dia. (mm)	Average Input Velocity Post Melt (m/s)	Post-Melt Temp Cavity Depth (mm)	Post-Melt Temp Cavity Dia. (mm)
5 (7)	245	66.65	15.84	228	59.81	13.78
5 (8)	242	57.81	12.52	222	71.41	12.69
5 (9)	244	58.91	12.52	230	72.50	13.78
10 (1)	226	58.18	12.15	236	46.04	15.23
10 (2)	229	61.12	12.15	230	59.09	14.5
10 (3)	246	61.49	N/A^3^	232	54.38	14.14
15 (4)	232	65.17	N/A^4^	230	58.36	16.31
15 (5)	241	58.18	12.15	226	59.09	13.78
15 (6)	247	67.02	16.94	224	56.19	13.41

^3^ Block 3 DoP measurement discounted due to concern around sabot influence and insufficient separation during flight

^4^ Block 4 DoP measurement discounted due to concern around accuracy of HSV measurement. Concern raised around surface finish of factory supplied block and ability to measure accurately

Contamination during melting and recasting was also considered but not witnessed, with the ball bearing and any sabot fragmentation carefully removed using a surgical scalpel before the melt cycles began. This was undertaken using the human eye, with no microscopy being utilised to confirm successful removal at a molecular level. Although minimised, the risk of introducing contamination to the material during the melting cycles is one which requires careful control. Metal flakes caused by fragmentation have the potential to act as nucleation sites for tearing or local stiffening, but they also have the potential to increase the rate of oxidative degradation [92, 93]. This study has utilised AISI 420 grade stainless steel ball bearings that have a reported hardness of 52–55 HRC Rockwell Scale to minimise against the potential for fragmentation [94] and as such concern was discounted for this study.

## Conclusions and Recommendations

An experimental campaign consisting of 18 shots against Perma-Gel which have been exposed to increasing number of melt/recast cycles has been undertaken to investigate the potential for this material’s performance to degrade within a ballistic setting. Depth of penetration measurements were taken pre and post melt cycle, alongside a comprehensive review of high-speed video footage to examine differences in cavity diameter maxima.

The main conclusions from this work are that careful consideration to the application of Perma-Gel within wound ballistics studies should be given due to notable degradation in depth of penetration (30.40% maximum observed difference), when utilising the parameters of this study. Additionally, assessment of temporary cavity indicates a 5.36% increase in maximum diameter post melt cycle further evidencing material degradation, for which it is proposed that a limit be placed on the number of melts cycles the material can undergo before disposal is required to mitigate against falsified data being produced.

Further, concerns remain surrounding its wider use, and the ability for its data to be easily referenced back to 10% gelatine or human cadavers. It is therefore recommended that researchers using Perma-Gel should:

- Calibrate each new batch/block with a reference projectile (steel BB or a projectile with well-known behaviour in 10% gelatine).
- Limit remelts - record number of melts; retire block after a conservative reuse threshold (establish threshold from pilot tests).
- Control temperature during testing and report it (block temperature and lab temperature).
- Run side by side tests with 10% gelatine or biological proxy for at least a subset of shots if you must claim clinical relevance.
- Document optical condition (clarity, discoloration, bubbles) that might affect imaging/interpretation.
- Document batch number, date received, number of recasts/melt cycles and each shot location for traceability.

To ensure the recommendations provided above are investigated, additional work should be undertaken on the environmental impact of incorrect disposal of Perma-Gel to explore severity as its constituent parts contain non-biodegradable materials such as mineral oil. Additionally, emphasis should be placed on undertaking additional mechanical property characterisation (density/viscosity/elasticity) for pre and post melt cycles focussing on microstructural changes by measuring mass/volume changes. Verification of rebound/elasticity tests (compression tests) should also be undertaken to observe recovery over time (melt cycle) with large changes indicating mechanical drift.

## Data Availability

Data supporting this study are openly available from the corresponding author on request.

## Appendix A

**Table 6:** Depth of Penetration and Velocity raw data

Serial 1	Depth of Pen. (mm)				Velocity (m/s)		
10 Bar Air	**Rule**	HSV rest	Avg.	HSV max depth	**Sensor**	HSV	Avg.
Block 1	**125**	139.1860	132	166.4345	**230**	222.4835	226
Block 2	**140**	149.4961	145	177.1124	**232**	225.7391	229
Block 3	**134**	152.4423	143	181.1628	**247**	244.0207	246
Block 4	**140**	159.8062	150	188.1589	**233**	230.2402	232
Block 5	**125**	133.2946	129	160.5426	**244**	238.3394	241
Block 6	**125**	141.7636	133	171.9574	**247**	247.2730	247
Block 7	**150**	160.9109	155	184.1089	**247**	242.3872	245
Block 8	**125**	134.7674	130	161.6473	**247**	237.5583	242
Block 9	**125**	141.3953	133	169.3798	**247**	240.7659	244
8 Bar Helium	**Rule**	HSV rest	Avg.	HSV max depth	**Sensor**	HSV	Avg.
Block 1	**163**	176.5377	170	203.0002	**244**	227.4540	236
Block 2	**142**	150.8002	146	181.9752	**238**	222.0129	230
Block 3	**153**	163.8506	158	188.5002	**238**	226.2537	232
Block 4	**169**	180.1627	175	204.8127	**238**	222.0129	230
Block 5	**150**	160.5877	155	187.4127	**233**	219.8330	226
Block 6	**138**	149.3502	144	171.4627	**233**	215.4804	224
Block 7	**143**	152.2502	148	177.6252	**235**	220.9213	228
Block 8	**142**	152.9752	147	177.2627	**230**	214.3922	222
Block 9	**140**	150.4377	145	173.6377	**238**	222.6624	230

## Footnotes

2 Cost correct as of March 2024.
